# A head-to-head comparison between two commercial software packages for hybrid dosimetry after peptide receptor radionuclide therapy

**DOI:** 10.1186/s40658-020-00308-9

**Published:** 2020-06-01

**Authors:** Daphne M. V. Huizing, Steffie M. B. Peters, Michelle W. J. Versleijen, Esther Martens, Marcel Verheij, Michiel Sinaasappel, Marcel P. M. Stokkel, Berlinda J. de Wit-van der Veen

**Affiliations:** 1 Department of Nuclear Medicine, Netherlands Cancer Institute, Plesmanlaan 121, 1066 CX Amsterdam, The Netherlands; 2 Department of Radiology and Nuclear Medicine, Radboud University Medical Center, Nijmegen, The Netherlands; 3 Department of Clinical Physics and Instrumentation, Netherlands Cancer Institute, Amsterdam, The Netherlands; 4 Department of Radiation Oncology, Radboud University Medical Center, Nijmegen, The Netherlands

**Keywords:** Dosimetry, PRRT, Quantification, Neuroendocrine tumours

## Abstract

**Background:**

Dosimetry after peptide receptor radionuclide therapy (PRRT) is increasing; however, comparing or pooling of dosimetric results can be challenging since different approaches are used. The aim of this study was to perform a head-to-head comparison of post-PRRT curve fitting and dosimetry obtained from two commercial software Hybrid Viewer Dosimetry and PLANET Dose.

**Methods:**

Post-therapy imaging included planar scintigraphy at 0.5, 4, 24 and 72 h post-injection of [^177^Lu]Lu-DOTA-TATE for kinetics and SPECT/CT at 24 h for quantification. On planar imaging, 2 cm regions-of-interest were positioned within the inferior pole of the kidneys and kidney cortex was segmented on low-dose CT. On both planar and SPECT/CT, 2 cm spheres were positioned in the proximal humerus (red marrow equivalent) and in the region with the highest uptake in tumour lesions. TACs were estimated with mono- and bi-exponential fits in both software systems, after which tissue absorbed (kidney, red marrow, tumour) and biological effective doses (kidney) were calculated. Agreement-ICC, Spearman correlation and Bland-Altman plots were used to compare results.

**Results:**

Mono-exponential fits showed the most comparable correlation between the measured and fitted data between both software. The ICC between absorbed dose outcomes was > 0.7 in tumour lesions and kidneys, but negative for the red marrow. Spearman correlation was > 0.9 for mono-exponential fits in kidneys and tumour lesions, and −0.7 in red marrow. Bi-exponential fits resulted in lower correlations and agreement values. Concordance between both software packages concerning the number of PRRT cycles with 7.4 GBq was observed based on a biological effective dose limit of 27 Gy to the kidneys.

**Conclusion:**

[^177^Lu]Lu-DOTA-TATE dosimetry results of two software packages were comparable in the same dataset, despite the limited number of imaging time-points. However, these results should be verified in a larger cohort before pooling of clinical data, as the obtained results will depend on acquisition protocol, timing and lesions definition.

## Background

Since the early 2000s, peptide receptor radionuclide therapy (PRRT) with [^177^Lu]Lu-DOTA-TATE is used for the treatment for neuroendocrine tumours (NET). In PRRT, a ‘one-size-fits-all’ treatment approach is most frequently applied [1]. So far, it is unknown what the optimal absorbed dose is to achieve a clinically meaningful therapy response without inducing toxicity in organs at risk, mainly the kidneys and red marrow. Personalized dosage prescription by assessing the mean absorbed dose in both tumour lesions and normal tissues based on [^177^Lu]Lu imaging data might improve PRRT response rates while preventing severe (grade 3-4) (sub) acute toxicity [2]. Multiple studies described dose relationships with tumour response and renal- and haematotoxicity [2–5]. Currently, absorbed dose limits for these organs are based on the extensive experience in external beam radiotherapy (EBRT) [6]. However, the biological effects of EBRT may not be directly transferable to PRRT, as EBRT dose delivery is geometrically highly focused and fractionated whereas PRRT includes kinetic behaviour of the radiopharmaceutical and continuous irradiation of targets [7].

Traditionally, dosimetry in radionuclide therapy is based on the world-wide accepted medical internal radiation dose (MIRD)-formalism using *S* values, assuming homogeneous tissue densities and radioactivity distributions, spherical tumours and reference man phantom organ geometries [8]. This methodology has been implemented in the OLINDA/EXM personal computer code in 2005 by Stabin et al. [9]. As dosimetry in radionuclide therapy is evolving past the status of a mere research tool towards clinical implementation, so are the software tools that can be used. Hence, the OLINDA/EXM-code has been commercialised by Hermes Medical Solutions (Stockholm, Sweden) into the FDA/CE-marked software. Additionally, a number of FDA/CE-marked voxel-based dosimetry methods using dose point kernels are nowadays available which uses the patient-specific organ and tumour geometries rather than phantom data to calculate absorbed doses at a voxel level [10]. These software tools generally provide user-friendly interfaces and operational stability, which allows for relatively easy incorporation into the clinical setting and the FDA/CE mark allows for clinical decision-making. Nevertheless, for clinically meaningful dose estimates the methodology of the entire dosimetry chain should be optimal, as is emphasised in the EANM guidance article by Gear et al. and in MIRD Pamphlet No. 26 [11, 12]. And although dosimetry procedures are generally performed with the best intentions, it is not always possible to comply with these guidelines due to clinical or logistic reasons.

In [^177^Lu]Lu-DOTA-TATE therapy dosimetry, a certain amount of radioactivity is administered to the patient and sequential post-therapy imaging is performed. The time-integrated activity in a specific target is determined by fitting the time-activity curve (TAC) derived from gamma camera images. Subsequently, conversion matrices are used to produce absorbed dose estimates. Variation in parameters, such as imaging time points, camera calibration, image acquisition parameters, target definition, TAC fitting, are all known to affect absorbed dose outcomes. With the increasing number of centres performing dosimetry, the use of different dosimetry workflows and software packages will inherently lead to variations in absorbed dose estimates, even when aspects such as patient preparation, imaging and calibration are harmonised. If these the discrepancies induced by software prove clinically relevant, translation of data across centres on for instance dose-limiting toxicities or absorbed tumour doses would become challenging. However, it is unknown whether dosimetry results from different (commercial) software packages could be pooled or used interchangeably. In nuclear cardiology for instance, the use of software to quantify cardiac function is common, but the results are not interchangeable and dedicated normal values have been derived for each software system [13].

In the current study differences between software packages are assessed with special focus on time-activity curve fitting and absorbed dose outcomes, given a standardised input of clinical [^177^Lu]Lu-DOTA-TATE imaging data. The input data includes imaging at four-time points, which is understandably not sufficient for bi-exponential fitting of the pharmacokinetic behaviour from a mathematical point of view. Still, this data does represent the clinical practise, and thus, the goal of this study was to compare to commercial software packages with a clinical dataset.

## Materials and methods

### Patients and PRRT treatment

This study includes ten consecutive patients treated with [^177^Lu]Lu-DOTA-TATE, with sufficient uptake (> liver) on [^68^Ga]Ga-DOTA-TATE PET/CT. Adequate renal, liver and haematological function were required and obstructions in renal flow were evaluated using ^99m^Technetium-MAG3 planar gamma imaging. Acceptable haematological parameter levels were haemoglobin ≥ 5.5 mmol/L, leucocytes counts ≥ 3.0 × 10^9^/L, neutrophil granulocytes counts ≥ 1.0 × 10^9^/L and platelet counts ≥ 75 × 10^9^/L. Serum eGFR should be ≥ 50 ml/min/1.7m^2^ and total bilirubin maximum three times the upper limit of normal. Patients had to stop long-acting somatostatin analogues (SSAs) at least 4 weeks and short-acting SSAs at least 24 h before each [^177^Lu]Lu-DOTA-TATE administration. The PRRT protocol included four cycles of 7.4 GBq [^177^Lu]Lu-DOTA-TATE, administered in 10-week intervals. For renal protection, an amino acid solution of 25 mg lysin and 25 mg arginine in 2 l of normal saline was infused in 4 h, starting 30-60 min before [^177^Lu]Lu-DOTA-TATE administration. In each patient, only one treatment cycle was used for analysis within this study to maintain independent measurements.

### Post-therapy imaging

Post-therapy [^177^Lu] Lu imaging included a hybrid workflow, with total-body planar imaging at 0.5, 4, 24 and 72 h after injection and one SPECT/CT of the thorax and abdomen after 24 h. All imaging was performed on a Symbia T2 (Siemens GmbH, Erlangen, Germany), equipped with a medium energy general purpose collimator. The primary energy window was positioned at 208 keV ± 10% with one downscatter (166.4–187.2 keV) and two general scatter windows (56.1–166.0 keV and 18.5–55.5 keV) for SPECT/CT reconstruction. The general scatter windows were used to obtain the total wide-spectrum counts according to the protocol designed by Beauregard et al. for this specific SPECT/CT system [14]. Total-body imaging was performed using both heads at 15 cm/min. SPECT acquisition parameters were non-circular, continuous rotations of both heads with 48 views of 13 sec/view per head. The SPECT image matrix size was 128 × 128 with 3.5 × 3.5 × 5 mm voxels. SPECT reconstruction included attenuation and scatter corrected 3DOSEM (FLASH3D) with 4 iterations and 8 subsets without post-reconstruction filtering or partial volume corrections. Regular quality control according to the vendor’s specifications was performed, and before each [^177^Lu]Lu-acquisition the energy spectrum and peaks were controlled. Local cross calibration between the SPECT/CT and VIK-202 dose calibrator (Comecer, Castel Bolognese, Italy) was performed using a homogeneous filled cylindrical phantom (9623 ml). This phantom was also imaged with the abovementioned settings. Though recovery coefficients were determined to optimize image reconstruction protocol and assess the effect of lesions size on quantification, no partial volume corrections were performed on any [^177^Lu]Lu gamma acquisitions in this study.

### Commercial dosimetry software

Segmentation, TAC fitting and dosimetric analysis was performed using hybrid viewer dosimetry module together with OLINDA/EXM v2.1 (Hermes Medical Solutions, Stockholm, Sweden) and PLANET Dose v3.1.2 (DOSIsoft SA, Cachan, France). Both software systems operate largely as a back-box, and only limited literature is available on their underlying assumptions and constraints.

In the hybrid viewer dosimetry module, the MIRD system is incorporated to estimate organ, lesion and whole-body mean absorbed doses, and at least three or four-time points are required to enable mono-exponential or bi-exponential fits, respectively. Curve fitting is performed in four steps: (I) extrapolation of the first imaging time points to *t* = 0, (II) trapezoidal integration from the first imaging time point to the first fit point which can be selected by the user, (III) bi- or mono-exponential fit using the Levenberg-Marquardt technique between the first fit time point and the last imaging time point and (IV) extrapolation of the curve created in the previous steps, unless the effective half-life is greater than the radionuclide half-life. In that case, the radionuclide physical half-life is used to fit the tail of the curve. Hybrid viewer dosimetry always requires geometric mean input from planar images. The integrated *S* values are previously calculated using Monte Carlo simulation with standard anthropomorphic phantoms derived from ICRP 89. The two kidneys are noted as one organ and if only one of the kidneys is indicated by the user, the paired organ option enables an estimation of the whole organ mean absorbed dose. The user can choose to work with the patient’s own organ and tumour masses, instead of the reference phantom organ masses, while assuming a density of 1 g/cm^3^.

PLANET Dose is complemented with the PLANET Onco platform, which includes the necessary contouring tools. Multiple planar scans should be manually registered and regions-of-interest (ROI) can be determined from either anterior/posterior views or using the geometric mean. The average number of counts in each ROI is used for kinetic input. Several curve fitting options are available, for example, mono-, bi- and tri-exponential and trapezoidal fits (with/without physical decay). The software relies on input from the user to select the proper fit-type and does not have any constraints. The absorbed dose is calculated on a voxel level using either voxel *S* value dose kernel convolutions or the local deposition method. Unlike OLINDA/EXM, organ and tumour masses are always CT-based from the individual patient. Three options are available for the location of *t* = 0. Origin forces the graph to go through (0,0), line indicates that the value of *t* = 0 is the same as the first imaging time point, and the continue option extrapolates the fit from the first imaging time point to *t* = 0. Again the user has to specify the desired option.

### Segmentation and dosimetry

All segmentations on both platforms were performed by one experienced viewer to achieve maximal comparability in target definition and delineation. Selected targets included both kidneys, red marrow and tumour lesions, the latter subdivided into ‘liver’ and ‘other locations’. Only tumour lesions with a diameter > 2 cm on diagnostic CT were selected to reduce the partial volume effects. Although these effects can have a major effect on count rate quantification, they have little effect on the direct comparison between the software packages as the error will be similar for both.

On each planar scintigraphy, circular ROIs with a diameter of 2 cm were drawn in the caudal part of the kidneys to minimise contribution of normal liver accumulation due to superimposition. Since the acquisition speed of all planar acquisitions is the same, average counts in the ROIs on the other time points were quantified proportionally to the planar scan at 24 h. The kidney cortex was segmented on the low-dose CT to determine the total uptake from SPECT; the CT-based kidney mass was used for analysis in both systems. Since OLINDA/EXM uses the average residence time of both kidneys for the average absorbed kidney dose, the average of the two mean absorbed doses in the kidneys calculated by PLANET Dose was used. To estimate red marrow uptake, ROIs and volumes-of-interest (VOIs) with diameter of 2 cm were located in the proximal humerus on planar and SPECT images, respectively. Although L2-L4 is often used as a surrogate for the red marrow uptake, a 2 cm diameter sphere was placed in the proximal humerus to minimise superimposition of (non-) physiological uptake from the intestines. According to ICRP 89, 2.3% of the total red marrow is presented in the upper half of the humerus and the volume of the proximal humerus volume is 180 ml based on the reference male phantom [15, 16]. The representative percentage of total red marrow in the VOI has to be entered in OLINDA/EXM, which is approximately 0.1% with a VOI volume of 4.2 ml [17]. This percentage was assumed the same for both male and female. By default, the red marrow in OLINDA/EXM is only a target organ. Since this is not possible in PLANET Dose, the red marrow was indicated as source organ in OLINDA/EXM. Red marrow and kidney ROIs were always drawn on the posterior view and copied to the anterior view to calculate the geometric mean.

As no consensus is available for tumour lesion delineation, a 2 cm sphere located in the region with the highest uptake was used to assess [^177^Lu]Lu-DOTA-TATE uptake in tumour lesions according to Del Prete et al. [3]. ROIs were drawn on either the posterior or anterior view depending on the highest observed uptake. A standard density of 1 g/cm^3^ was assumed for the sampled lesions in OLINDA/EXM [18]. For optimal comparison between OLINDA/EXM and PLANET Dose, the geometric mean (GM) was determined for all planar ROIs as this is required in OLINDA/EXM.

TACs were fitted using bi- and mono-exponential functions in both software systems, as these functions have been used to describe the pharmacokinetic behaviour of somatostatin analogues [1, 19, 20]. For TAC fitting, EANM guideline on dosimetry reporting states the use of three imaging points per phase, still only four points were acquired in this study due to patient logistics [21]. We acknowledge that fitting of two exponential functions with only four-time points is mathematically erroneous and for example, a trapezoidal integral with physical decay from the last data point could be a better option. However, OLINDA/EXM does not provide the trapezoidal fit and since the goal of this study is to compare the software in their current form, only mono- and bi-exponential fitting was applied. TACs were integrated according to the default setting in both software packages; until 2400 h after injection in PLANET Dose and until infinity in OLINDA/EXM.

Neither software package provides calculation of the BED, a parameter based on logarithmic cell kill to account for the biological effect of dose rate [22]. The BED was calculated manually using fit results from both software for kidneys only since the most evidence and experience in literature is for this organ and far less for tumour lesions and red marrow. The following formula derived from MIRD pamphlet No. 20 was used [22]:

1 $$ BED=\frac{R_0}{\lambda_e}\left[1+\frac{R_0}{\left(\mu +{\lambda}_e\right)\left(\alpha /\beta \right)}\right] $$

with *R*_0_ as the initial dose rate (mGy.MBq^−1^.s^−1^), *λ*_e_ as the target-specific effective decay constant (h^−1^), *μ* as the sublethal damage repair rate (h^−1^) and *α*/*β* according to the linear-quadratic model. For this study, values for *μ* and *α*/*β* for kidneys were chosen according to literature [23, 24]: *μ* = 0.248 h^−1^ and *α*/*β* = 2.6 Gy.

### Analysis and statistics

Absolute (in Gy/GBq) and relative (%) mean absorbed dose differences between OLINDA/EXM and PLANET Dose were assessed using both Bland-Altman analysis and correlation measures. Bland-Altman plots provide a visual interpretation of the difference between two measurements with regard to the average value of the two, as well as a systematic mean difference between all measurements (bias) and the corresponding standard deviation. The 95% limits of agreement (LOA) were used to indicate whether the observed variation could be clinically relevant [25]. Spearman’s rank correlation and interchangeably correlations (absolute agreement, with two-way mixed effects and single rater assumptions) were determined of all fits per location: kidneys, red marrow, and tumour lesions. Since OLINDA/EXM uses the average residence time of both kidneys for the absorbed dose calculation, the average of the two mean absorbed doses in the kidneys calculated by PLANET Dose were used for comparison.

Biological effective doses (BED) were calculated for all patients using Equation 1 according to MIRD pamphlet no. 20. The absorbed doses of the first cycle were used to estimate the cumulative kidney dose over four treatment cycles, and compared to the BED thresholds for kidneys of 27 G to determine the clinical impact of differences between software packages [23, 24].

## Results

**Fig. 1:**
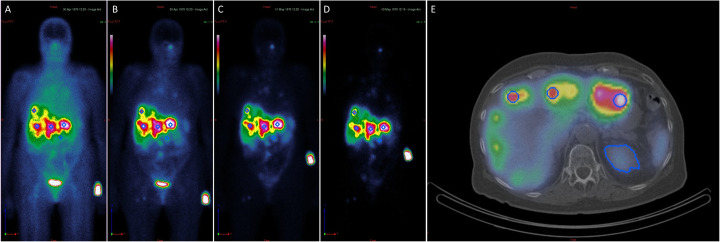
Example of ROI and VOI drawings in PLANET Dose. **a**-**d** Anterior planar imaging on all four time points. **e** SPECT/CT after 24 h

All ten included patients were diagnosed with a histological confirmed disease: six patients with a NET grade 1, three patients with NET grade 2 and one patient with a metastatic medullary thyroid carcinoma. Patient characteristics are shown in Table 1. In total, 19 ROIs were located in the kidneys (one kidney was not visible on planar imaging), 10 in the red marrow and 28 in tumour lesions (20 liver lesions and 8 other locations). A segmentation example is shown in Fig. 1.

**Table 1:** Patient characteristics

	1	2	3	4	5	6	7	8	9	10
Age^†^ (year)	69	74	57	45	74	63	76	68	73	71
Gender (M/F)	M	M	F	M	M	F	F	M	F	F
Primary	Lung	Pancreas	Ileum	Ileum	Unknown	MTC*	Ileum	Pancreas	Unknown	Ileum
Grade	2	1	1	2	1	NA	2	1	1	1
MIB-1	3-20%	2%	1%	1%	1%	NA	5%	2%	1%	2%
Ki-67	NA	2-5%	3%	2%	0	NA	5%	0%	0%	1%
Resection primary	Y	N	Y	Y	N	Y	N	N	Y	Y
Liver embolization	N	Y	Y	Y	N	N	N	N	N	N
SSA therapy	Y	Y	Y	Y	Y	N	N	Y	Y	Y
Chemo/targeted therapy	N	N	N	N	N	N	N	Y	N	N
Metastatic sides	Liver, LN, bone, other	Liver, LN, bone	Liver, LN, bone	Liver, other	Liver, LN, bone, other	LN	Liver, LN, bone, other	Liver, LN, other	Liver, LN, bone, other	Liver, LN, bone, other
Tumour lesions included	None	Liver: 3 Other: 1	Liver: 2 Other: 1	Liver: 2	Liver: 2 Other: 2	Other: 1	Liver: 4 Other: 1	Liver: 5 Other: 1	Liver: 1 Other: 1	Liver: 2
Administered activity (MBq)	7131	7253	7176	7271	7188	7212	7613	7476	7338	7373

*Medullar thyroid carcinoma

*LN* lymph nodes; *NA* not available; *SSA therapy* somatostatin analogue use during PRRT treatment

*†Age at start PRRT

### Time-activity curve fitting

**Fig. 2:**
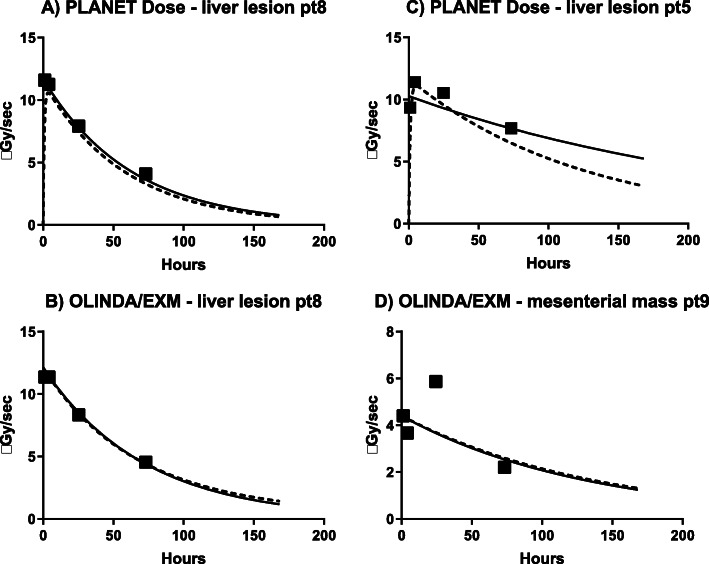
Examples of mono-exponential (solid line) and bi-exponential (dashed line) fits of tumour lesions with OLINDA/EXM and PLANET Dose. (**a**-**b**) Present good mono-exponential fits, whereas (**c**-**d**) show suboptimal fittings

**Fig. 3:**
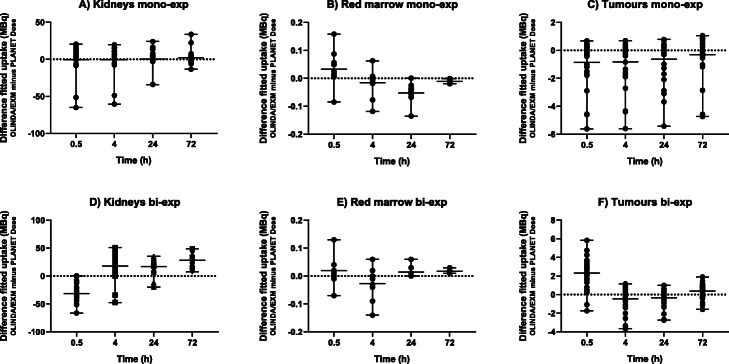
Absolute difference in fitted value of [^177^Lu]Lu-DOTA-TATE uptake between OLINDA/EXM and PLANET Dose in the same targets, after mono-exponential (mono-exp) and bi-exponential (bi-exp) fitting. The mean difference and range are shown and the dotted line represents no difference between the two software packages

Table 2 shows the Spearman’s rank correlation (rho) results between the measured and fitted data points for the TAC fits. As PLANET Dose was able to determine the bi-exponential ‘line’ fit for only 2 kidneys and for one red marrow, these results were not used for further analysis. The limited number of time points used in this study induced inaccurate bi-exponential fits, especially profound in PLANET Dose. However, this is considered a limitation of the input data, and not of the software. Figure 2 shows examples of fits using both mono-and bi-exponential functions. Visually, the data points in Fig. 2c would be better fitted with a bi-exponential function than with a mono-exponential function. In Fig. 3, the absolute differences in [^177^Lu]Lu-DOTATATE uptake are shown for both mono- and bi-exponential fitting for all targets. Note that despite the fact that the input acquisitions are similar and that the process of delineation was standardised, still there is a relatively large range on the difference in calculated [^177^Lu]Lu-DOTATATE uptake.

**Table 2:** Spearman’s rank correlation (rho) of all TAC-fits

			Kidney	Red marrow	Tumour lesions		
					All	Liver	Other
Mono-exponential	OLINDA/EXM		0.776	0.905	0.906	0.917	0.880
	PLANET Dose	Line	0.917	0.910	0.938	0.935	0.946
		Origin	0.916	0.874	0.931	0.927	0.941
Bi-exponential	OLINDA/EXM		0.913	0.860	0.917	0.920	0.909
	PLANET Dose	Line	-	-	0.973*	0.984*	0.849*
		Origin	0.737	0.943	0.728	0.761	0.647

*Could be calculated for 2/3 of the data, therefore excluded from the analysis

### Mean absorbed dose values

**Fig. 4:**
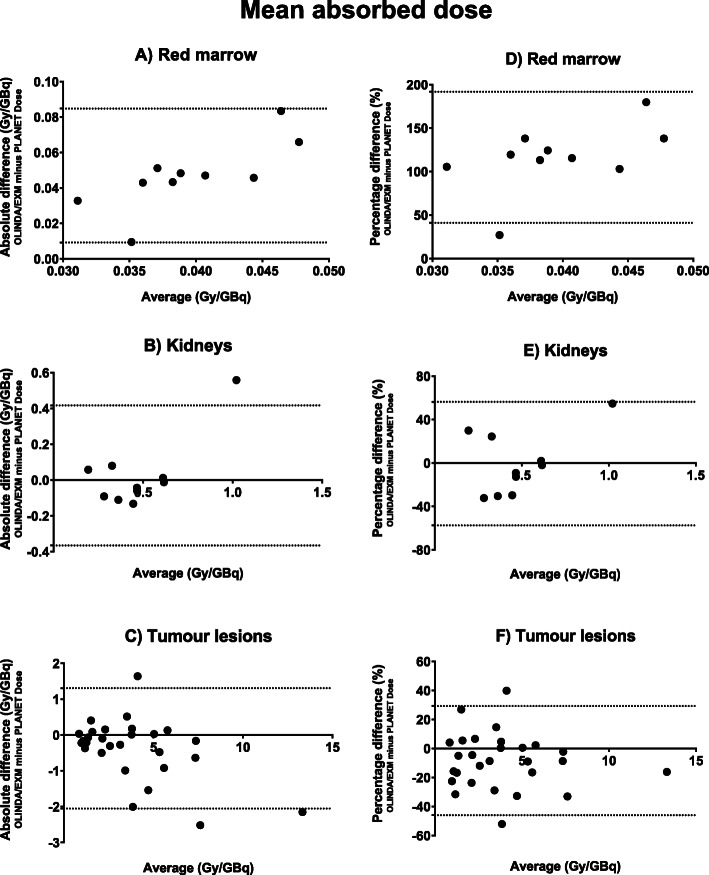
Mean absorbed dose difference and average between OLINDA/EXM and PLANET Dose determined using mono-exponential fitting. Note that both absolute values (**a**-**c**) and relative differences (**d**-**f**) are provided. Relative differences are calculated using the following equation: $\frac{OLINDA/ EXM- PLANET\ Dose}{average}\times 100\%$

**Fig. 5:**
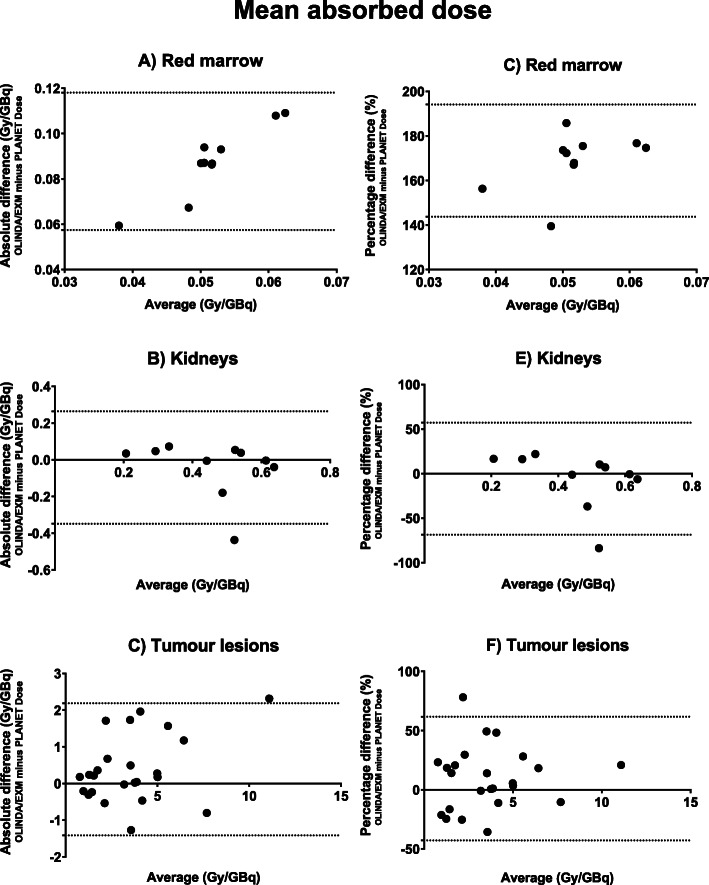
Mean absorbed dose difference and average between OLINDA/EXM and PLANET Dose using bi-exponential fitting. Note that both absolute values (**a**-**c**) and relative differences (**d**-**f**) are provided. Relative differences are calculated using the following equation: $\frac{OLINDA/ EXM- PLANET\ Dose}{average}\times 100\%$

Mean absorbed doses for kidneys and red marrow are provided in Table 3 and for tumour lesions in Table 4. Bland-Altman plots are provided in Figs. 4 and 5 for both mono- and bi-exponential fitting, respectively. Bland-Altman analysis results, ICC and Spearman correlation between both software are shown in Table 5. Note that the absorbed dose for red marrow calculated with OLINDA/EXM is at least twice that of PLANET Dose (Fig. 4). Such a large difference is not observed for the kidney doses, though it seems that the data is mainly influenced by one data point in the mono-exponential data (patient #6). After exclusion of this single outlier, the kidney bias reduced to −0.033 ± 0.074 Gy/GBq [95% LOA −0.18-0.11] with a correlation of 0.876. For tumour lesions, there is a large variability in uptake amongst the patients to begin with, the differences between the software packages on the other hand is relatively small. When comparing the results of the mono- and bi-exponentially fitted data in tumour lesions, there is a very good correlation between the calculated absorbed doses (*r* = 0.915, *r*^2^ = 0.836).

**Table 3:** Mean absorbed dose (Gy/GBq) per target location of all patients

	Mono-exponential				Bi-exponential			
	Kidneys		Red marrow		Kidneys		Red marrow	
Patient	O/E	PD	O/E	PD	O/E	PD	O/E	PD
1	0.61	0.62	0.063	0.015	0.61	0.62	0.095	0.008
2^‡^	0.37	0.29	0.088	0.005	0.37	0.30	0.098	0.004
3	0.31	0.43	0.067	0.022	0.44	0.44	0.115	0.007
4	0.24	0.33	0.048	0.015	0.32	0.27	0.094	0.007
5	0.22	0.16	0.063	0.012	0.22	0.19	0.010	0.007
6	1.30	0.74	0.060	0.017	0.30	0.74	0.095	0.009
7	0.44	0.49	0.081	0.015	0.56	0.52	0.117	0.008
8	0.38	0.52	0.058	0.015	0.40	0.58	0.094	0.007
9	0.62	0.61	0.064	0.017	0.62	0.66	0.068	0.008
10	0.44	0.50	0.040	0.030	0.55	0.50	0.082	0.015

*O/E* OLINDA/EXM; *PD* PLANET Dose

^‡^This patient had one visible kidney on planar imaging

**Table 4:** Mean absorbed dose (Gy/GBq) per lesion of all patients

		Mono-exponential		Bi-exponential	
Patient	Location	OLINDA/EXM	PLANET Dose	OLINDA/EXM	PLANET Dose
2	Liver	4.94	3.29	*	
2	Liver	2.10	2.20		
2	Liver	2.85	4.85		
2	Other	5.85	5.72		
3	Liver	5.05	5.02	5.05	3.09
3	Liver	1.63	1.54	1.92	1.56
3	Other	1.15	1.36	1.64	1.43
4	Liver	7.02	7.66	3.78	3.75
4	Liver	3.78	3.26	7.02	5.85
5	Liver	12.26	14.40	12.25	9.93
5	Liver	6.35	8.87	6.35	4.79
5	Other	3.89	3.71	3.89	3.85
5	Other	2.42	2.72	4.38	2.65
6	Other	1.71	1.31	3.05	1.33
7	Liver	5.10	5.57	5.10	4.92
7	Liver	5.13	6.05	5.13	4.85
7	Liver	7.29	7.45	7.29	8.09
7	Liver	3.94	5.48	3.94	4.40
7	Other	0.99	1.16	1.40	1.16
8	Liver	3.78	3.77	3.78	3.29
8	Liver	2.38	2.23	2.62	1.94
8	Liver	2.93	3.92	2.93	4.20
8	Liver	0.99	1.36	1.09	1.40
8	Liver	0.86	1.36	0.86	0.68
8	Other	3.00	3.28	3.18	3.20
9	Liver	0.87	1.09	0.87	1.07
9	Other	3.00	3.27	1.86	2.39
10	Liver	1.31	1.38	1.31	1.55

*The dosimetric results of this patient were not reliable (very high dose values)

**Table 5:** Comparison of the dosimetry outcomes

			Kidneys	Red marrow	Tumour lesions
Mono-exponential	Absolute difference (Gy/GBq)	Bias	0.026 ± 0.12	0.047 ± 0.019	−0.37 ± 0.86
		95% LOA	−0.37 − 0.42	0.01 − 0.08	−2.05 − 1.31
	Relative difference (%)	Bias	−0.53 ± 29.03	116 ± 38.5	−8.36 ± 19.19
		95% LOA	−57.4 − 56.4	41.0 − 192	−46.0 − 26.3
	Spearman correlation		0.903	−0.701	0.963
	ICC - agreement		0.708	Negative	0.804
Bi-exponential	Absolute difference (Gy/GBq)	Bias	−0.042 ± 0.16	0.088 ± 0.015	0.39 ± 0.92
		95% LOA	−0.348 − 0.265	0.057 − 0.118	−1.41 − 2.19
	Relative difference (%)	Bias	−5.63 ± 32.07	169 ± 12.8	−9.52 ± 26.66
		95% LOA	−68.5 − 57.2	144 − 194	−42.7 − 61.8
	Spearman correlation		0.491	−0.418	0.907
	ICC - agreement		0.537	Negative	0.921

*LOA* limits of agreement, *ICC* intraclass correlation coefficient

### BED and clinical implications

**Fig. 6:**
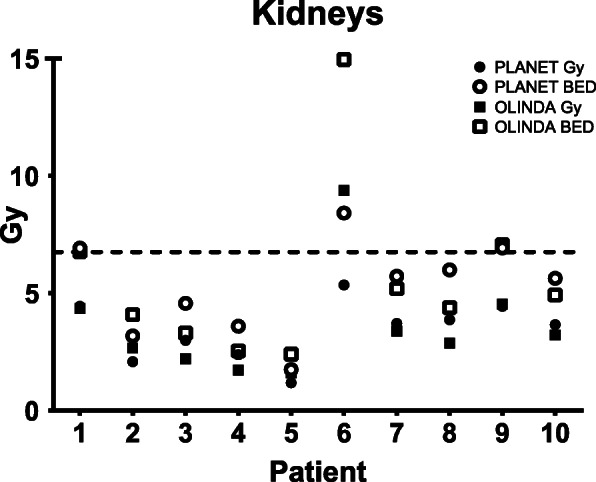
Mean absorbed dose and BED in the same target. The dotted line represents the BED threshold for one cycle (6.75 Gy) to allow for the maximum of 27 Gy BED

Figure 6 shows the results for estimated absorbed doses and BED per patient for one cycle for the kidneys. A total of three patients exceeded the 27 Gy threshold limit after four cycles of 7.4 GBq for both dosimetry software (patient #1, 6 and 9 for both software) while assuming four times the BED to the kidneys as determined in this therapy cycle.

## Discussion

In the current study differences in estimated mean absorbed dose calculated by two commercially available software packages was determined on clinical [^177^Lu]Lu-DOTA-TATE imaging data. As described in the EANM practical guidance on uncertainty analysis for molecular radiotherapy, many factors affect the absorbed dose calculation, and thus the comparison between two software systems [11]. Though the study results indicate that the dosimetry outcomes from software packages could not directly be used interchangeably, the clinical impact of the found differences has to be placed in relation to other uncertainties, such as acquisition protocol, delineation, and TAC-fitting.

### Differences between software packages

One objective of this study was to harmonise as many parameters (same patient population and scans, ROI/VOI drawings) between both software packages, and to evaluate the available dosimetry options and outcomes. For this reason, the convolution with density correction was selected for PLANET Dose. Convolutions without density corrections and the local deposition method are also available in this software package; however, in our opinion convolution and density correction provides the most sophisticated dosimetry calculation. For OLINDA/EXM, a minimum number of three or four imaging time points is required for TAC fitting using a mono-exponential or bi-exponential approach, respectively. In contrast, a minimum number of time points is not specified in PLANET Dose. On the other hand, PLANET Dose provides a mean square deviation and rho values of the fit, which support the user to select the most optimal fit for specific targets. Such assistance for curve fitting is not provided in OLINDA/EXM.

Since red marrow and kidneys are considered as main organs at risk in PRRT, no other organs were taken into consideration for dosimetry analysis. In OLINDA/EXM, both kidneys are considered as one organ. However, the mean absorbed dose of the left and right kidney can be different according to the results of PLANET Dose. In this study, the differences between the two software packages were evaluated using the CT-based kidney mass to increase comparability between the two. With respect to red marrow dosimetry, it is advised to take the bone and the remainder of the body into consideration using the MIRD formulism [26]. Since it is not possible to model these compartments in PLANET Dose, only the self-dose within the red marrow was assumed in OLINDA/EXM.

Multiple factors could contribute to the differences in dosimetric outcomes between the two software packages. The difference in curve fitting approach and assumptions is likely to have a major influence, however, exact differences cannot be pointed out due to the vendors’ black box. Nonetheless, one could note that bi-exponential fits of OLINDA/EXM started at the highest observed dose rate, which is similar to the ‘line’ option of PLANET Dose. However, PLANET Dose was not able to fit all targets with the bi-exponential ‘line’ option. Therefore, the assumption that bi-exponential fit should start at (0,0) seems anchored in PLANET Dose, which is not an option in OLINDA/EXM. In addition, the dosimetric approach using organ-based dosimetry (MIRD) vs. voxel-based dosimetry is fundamentally different. *S* values are fixed per tissue type whereas the voxel-based approach is independent of analysed tissue. The total contribution to the difference between OLINDA/EXM and PLANET Dose is a sum of all mentioned parameters, with various weightings depending on the tissue type and kinetic properties.

In on-going clinical trials, the BED is used to indicate the number of treatment cycles suitable for an individual patient [27]. For vendors, it is highly recommended to implement the possibility to calculate BED in future software updates. From a clinical point of view, both software packages showed agreement in terms of which patients would exceed the 27 Gy BED limit for the kidneys, assuming four times the BED as determined in the therapy cycle used in this analysis.

Additional comparisons with other dosimetry software tools would further contribute to the collaboration between centres on this topic. In this study, no evaluation with absorbed dose estimates calculated using *S* values from open-source data (e.g., IDAC Dose) or in-house software was performed. In general, these calculations often rely on time-integrated activity as input. As mentioned before, this TAC fit has major contributions to the final absorbed dose estimate and these open-source programs cannot be used clinically as they are not FDA/CE approved. The goal of this study was to compare TAC fits and absorbed dose estimates from commercially available software packages to stimulate the clinical implementation and cooperation between centres for post-PRRT dosimetry.

### Image acquisition and timing

[^177^Lu]Lu has two main photopeaks at 113 keV and 208 keV available for gamma imaging. In this study, the 208 keV photopeak was selected because it has the highest yield (~ 10%), as recommended by the EANM/MIRD pamphlet no. 26 [12]. In the acquisition protocol applied in this study, the counts in the 113 keV photopeak are used to determine the total-wide spectrum counts as suggested by Beauregard et al. [14]. The main drawback of the current study is the limited number and timing of the imaging time points, disabling correct fits according to mathematical and biological requirements [28]. In clinical practise, however, it is inconvenient for patients to undergo six post-therapy scans; therefore, we opted for four-time points. Especially, multiple late imaging time points after discharge from the hospital are not considered patient-friendly. The addition of one late time point (> 4 days post-injection) would have improved the estimation of the tail of the TAC. One could argue that the addition of an extra virtual time point at approximately 1 week after administration could improve the fits. However, this virtual time point would have been chosen based on the currently estimated TAC-fit, and hence, has limited impact on the data. On the other hand, mono-exponential fitting is generally considered a safe choice when limited number of data points, acquired within the effective half-life time of ^177^Lu-DOTATATE (~ 4 days), are available [28, 29].

### Target segmentation

Currently, no consensus or standardised method for lesion segmentation on [^177^Lu]Lu-DOTA-TATE SPECT/CT imaging is available. In order to deal with partial volume effects, image correction based on recovery coefficients is proposed by Finocchiaro et al. [30]. In the current study, this correction was not performed as the size of the ROIs and VOIs was fixed at 2 cm diameter, according to Del Prete et al. [3], resulting in the same partial volume effects. Partial volume correction would affect the dosimetry outcome; however, the standardized volume segmentation is suitable for dosimetry software comparison. In addition, the diameter of lesions included for analysis was > 2 cm on diagnostic CT. Due to the sampling methodology on post-therapy imaging, the lesion size was not deemed necessary and therefore not measured. Next to that, all ROIs and VOIs were drawn once by one person, preventing intra-observer variability in delineation.

Estimation of the red marrow absorbed dose with the method used in this study will inherently depend on the position of the ROI. Both the ROI placement by the user on planar images and the relatively high image noise signal in the proximal humerus area will affect the TAC. A common method for red marrow dosimetry is based on segmentation of the lumbar vertebrae [19]. However, in [^177^Lu]Lu-DOTA-TATE planar acquisitions it is difficult to correctly locate a ROI in L2-L4 without any superimposition of physiological or tumour uptake. Since physiological uptake in the upper arm is limited, the humerus was selected to represent the red marrow.

The geometric mean images were used in the current study since OLINDA/EXM only allows for this type of input. For the kidneys, located dorsally in the body, higher uptakes were observed on the posterior planar view alone, while the position averaged GM inherently introduces physiological counts from the intestines, spleen or abdominal lesions. The use of a GM for quantification generally reduces the effect of patient positioning with respect to the camera head, whereas a non-GM approach lowers the contribution of uptake in other tissue. This influence of normal tissue uptake is a reason to opt multiple time point SPECT/CT when the liver or abdominal lesions are of main interest.

### Time activity curve fitting and time-integrated activity calculation

As in all pharmacological evaluations, fitting of the data has a large effect on the calculation of the area under the curve, and hence, on the estimated absorbed dose. In MIRD pamphlet no. 16 is stated that for each ‘phase’ three-time points are needed, so for bi-exponential fits six imaging time points would have been the minimum for an accurate fit [31]. So, it is undesirable to fit four data points with a bi-exponential fit from a pure mathematical point of view, as this will inherently result in an unreliable model given the noise in the data. Trapezoidal integration with physical decay from the last time point would have been a better option, because the integral is less prone to fitting constraints that come with mono- and bi-exponential fit functions. Furthermore, trapezoidal integration would also benefit from an additional late imaging time point to improve estimation of the tail of the curve by postponing the assumption of physical decay later in time. OLINDA/EXM does not provide the option for trapezoidal fitting, consequently, this fitting option was also not applied in PLANET Dose. The scope of the study was to compare two software packages; therefore, the fitting procedures were standardized when possible. Still, it would be desirable to known which constraints are implemented within the software for each fitting option in the dosimetry software.

The mono-exponential fit provided the most comparable correlation coefficient for kidneys, red marrow and tumour between the software systems in this study. In bi-exponential fitting, PLANET Dose visually often failed to fit the peak of the curve correctly, thus resulting in a large underestimation. Still, even when the fit seemed inaccurate both software packages do provide absorbed dose outcomes, and interpretation of this data is left at the decision of the user. The overall estimated absorbed doses based on mono- and bi-exponentially fits in our study, showed a good correlation. More explicit differences between the uptake determined by OLINDA/EXM and PLANET Dose were observed in the early time points (before 24 h). Also, the correlation between the measured data and the estimated data at these time points according to the fit was suboptimal as shown in Table 2. All rho values were below 0.95 for each of the target locations, both for mono- and bi-exponential. According to Guerriero et al. a mono-exponential fit is safe if data after 24 h up to two effective half-lives is available. Bi-exponential fitting could be performed if data up to 2-3 days are available and if relevant pharmacokinetics occurs in the first day. They showed that based on these requirements and five-time point imaging, the time-integrated activity ratio is 1.02 ± 0.22 for mono- and 1.02 ± 0.07 for bi-exponential [28]. These values suggest that on average mono- and bi-exponential fits have similar results for [^177^Lu]Lu-DOTA-TATE; however, bi-exponential fits have better agreement with a lower standard deviation. The question is whether these differences have a significant influence on the final dosimetric, as many other factors like camera calibration and lesion delineation also play an important role. The time-activity curve fit uncertainty can also be different between localisations of tumour lesion; for example, 26% for a liver lesion and 0.1% for a pancreatic lesion after mono-exponential fitting [11]. Nevertheless, in this study certain cases showed a clear visual deviation from the measured data points. For example in the kidneys of patient #6, which is the outlier in the mono-exponential fit in Fig. 2. Review on the kinetics of these kidneys shows that a bi-exponential fit would have been more suitable.

For clinical implementation of dosimetry, reduction of the number of imaging time points would be desirable for patients and clinics. Though, on the other hand, the quantitative error should be within acceptable ranges. Hänscheid et al. proposed the use of one quantitative imaging time point at 4 days after injection assuming mono-exponential decay, which resulted in a quantitative error < 10% [29]. This method works for targets with an effective half-life between 38 and 128 h.

### Future work

In the current study, no associations with clinical outcomes were investigated. Ultimately, the relation between dosimetric analysis and clinical outcomes is essential in order to implement dosimetry in personalized radionuclide therapy planning. The first step towards the use of dosimetry in a clinical setting is consensus on methodology. In this study, we showed that there are slight differences in outcomes achieved by different software systems. However, when taking the many observed uncertainties for radionuclide therapy dosimetry analysis into account [22], the differences between both approaches are negligible.

The current results should be verified in a larger cohort, since this dataset includes a limited number of patients with certain tumour characteristics. Most importantly, the TAC fitting should be performed including later time points, such as 168 h after administration, for improved estimation of the tail of the curve. For patient comfort and nuclear medicine department logistics, the total number of time points should not be increased. Instead, early time points could be replaced by late time points when assuming mono-exponential decay since early time point result in an underestimation of the mean absorbed dose [32]. Post-therapy imaging at ~ 5, 24 and 168 h post-injection should be clinically feasible and enable time-activity curve fits with trapezoidal fit and physical decay from the last time point. Finally, cross-calibration of [^177^Lu]Lu between centres and a standardized (NIST) source would further improve comparability of [^177^Lu]Lu SPECT/CT quantification.

## Conclusion

Post-therapy [^177^Lu]Lu-DOTA-TATE dosimetry results of two software packages were comparable in the same dataset derived from a limited number of patients. Nevertheless, it is not advised to use these software packages interchangeably in the clinical setting or in a study, as there are differences in methodology. Additionally, the obtained results will depend on acquisition protocol, timing, and pharmacological behaviour of the radiopharmaceutical.

## Data Availability

The datasets used in this manuscript are available from the corresponding author on reasonable request.

## Abbreviations

CE: Conformité Européenne
CT: Computed tomography
EBRT: External beam radiotherapy
eGFR: Estimated glomerular filtration rate
FDA: Food and Drug Administration
GBq: Giga Becquerel
GM: Geometric mean
Gy: Grey
Hb: Haemoglobin
LOA: Limits of agreement
ICC: Intraclass correlation coefficient
MAG3: Mercaptoacetyltriglycine
MIRD: Medical internal radiation dose
NET: Neuroendocrine tumour
OSEM: Ordered-subset expectation maximum
PET: Positron emission tomography
PRRT: Peptide receptor radionuclide therapy
ROI: Region-of-interest
SSA: Somatostatin analogues
SPECT: Single photon emission computed tomography
TAC: Time activity curve
VOI: Volume-of-interest
